# Co-treatment With Everolimus, an mTOR-Specific Antagonist, or Downregulation of ELK1 Enhances the Sensitivity of Pancreatic Cancer Cells to Genistein

**DOI:** 10.3389/fcell.2021.633035

**Published:** 2021-09-03

**Authors:** Tianyu Li, Tiantao Kuang, Zhaoshuo Yang, Qiqi Zhang, Wen Zhang, Yue Fan

**Affiliations:** ^1^Department of Integrated Traditional Chinese Medicine and Western Medicine, Zhongshan Hospital, Fudan University, Shanghai, China; ^2^Department of General Surgery, Zhongshan Hospital, Fudan University, Shanghai, China; ^3^Cancer Center, Zhongshan Hospital, Fudan University, Shanghai, China

**Keywords:** pancreatic cancer, genistein, Elk1, DEPTOR, mTOR, everolimus

## Abstract

Genistein is a natural isoflavone with pharmacological or potentially anti-tumor properties. However, the resistance of cancer cells to genistein remains a major obstacle. This study focused on the mechanism implicated in the resistance of pancreatic cancer (PC) cells to genistein and the mechanism of action. First, key molecules and signaling pathways related to genistein resistance in PC cells were explored using bioinformatics tools. DEP domain containing MTOR interacting protein (DEPTOR), a typical inhibitor of the mammalian target of rapamycin (mTOR) signaling, was predicted to be poorly expressed in the genistein-resistant PC cells. Thereafter, genistein-resistant PC cells (Panc-1 and PaCa) were constructed. Altered expression of DEPTOR was introduced in cells, and everolimus (ELM), an mTOR-specific antagonist, was administrated in cells as well to examine their roles in genistein resistance. The cell apoptosis was examined *in vitro* and *in vivo* in mouse xenograft tumors. The upstream regulator of DEPTOR was predicted *via* bioinformatic tools. The bioinformatic analyses showed that the PI3K/AKT/mTOR signaling pathway was activated in the setting of DEPTOR downregulation in genistein-resistant PC cells. DEPTOR overexpression reduced the 50% inhibiting concentration (IC50) of genistein in PC cells and suppressed mTOR phosphorylation, and it increased caspase-3 activity, LDH release and apoptosis in PC cells. ELM treatment enhanced the sensitivity of PC cells to genistein *in vitro* and it strengthened the tumor-eliminating role of genistein in mice. ETS transcription factor ELK1 (ELK1), a transcription factor that negatively regulated DEPTOR transcription, was suppressed by genistein. Upregulation of ELK1 suppressed DEPTOR transcription and reduced the genistein sensitivity of cells, and it also blocked the genistein-sensitizing roles of ELM in PC cells. In conclusion, this study demonstrated that ELK1 reduces DEPTOR transcription, leading to mTOR phosphorylation and the drug resistance of PC cells.

## Introduction

Pancreatic cancer (PC) is a highly malignant tumor with an estimated 459,000 cases diagnosed and 432,000 deaths worldwide in 2018, which ranks the seventh leading cause of cancer death in both genders because of the poor prognosis (Bray et al., 2018). PC is usually diagnosed due to the patient seeking medical help for abdominal pain (Lohse and Brothers, 2020). Due to the lack of symptoms, patients are often diagnosed with local or distal metastasis that are not eligible for surgical resection, and the 5-year survival rate of these patients was less than 9% (Siegel et al., 2019). Despite better understanding in the biology and pathogenesis of PC, current therapeutic regimens are still insufficient, and the chemoresistance is the major obstacle in PC prognosis (Grasso et al., 2017). In addition to an early diagnosis, developing novel options for PC treatment or battling the resistance of PC to chemotherapies are important issues for PC management.

Genistein is a soy-derived isoflavanoid compound that showed pharmacological or anti-oncogenic properties in cancers by regulating cell apoptosis, cell cycle, and angiogenesis and suppressing metastasis (Spagnuolo et al., 2015; Mukund et al., 2017). Genistein (or its analog) has been reported as a candidate anti-tumor regimen for PC treatment and has shown significant anti-proliferative and pro-apoptotic functions in PC cells in a dose-dependent manner (Xia et al., 2014; Lohr et al., 2016; Bi et al., 2018). However, PC cells may easily develop resistance to genistein (Subramaniam et al., 2018). In the current study, the integrated bioinformatics suggested that DEP domain containing MTOR interacting protein (DEPTOR), a typical inhibitor of the mammalian target of rapamycin (mTOR) signaling, was poorly expressed in the genistein-resistant PC cells, which led to the activation of the phosphatidyl inositol 3-kinase/protein kinase B/mTOR (PI3K/Akt/mTOR) signaling pathway. Moreover, ETS-domain containing protein (ELK1), a downstream transcription factor of the epidermal growth factor receptor (EGFR), was predicted to own binding sites with DEPTOR. The PI3K/Akt/mTOR pathway is frequently dysregulated in human cancers, and mTOR is the master regulator of this pathway that has a critical role in cancer and participate in cell transformation, growth, survival, and drug sensitivity (Murugan, 2019). In addition, the mTOR activation has been associated with increased drug resistance in cancers (Baumann et al., 2017; Yao Y. et al., 2019). The EGFR has been demonstrated to show a negative correlation with DEPTOR and activated mTOR phosphorylation in human cancers (Zhou et al., 2016). Therefore, we speculated that genistein may suppress EGFR in cancer cells. The failure in EGFR suppression might lead to DEPTOR downregulation and mTOR activation, which is responsible for the development of resistance of PC cells to genistein.

Everolimus (ELM) is a commonly used antagonist of mTOR that has shown tumor-suppressing functions in cancer management (Falkowski and Woillard, 2019). Interestingly, co-treatment of ELM with imatinib was found to reduce the imatinib resistance in chronic myeloid leukemia (Alves et al., 2019). In addition, ELM was found to overcome the gemcitabine resistance of PC cells and induce cell apoptosis (Peng and Dou, 2017). Whether the administration of ELM similarly increases the sensitivity of PC cells to genistein remains unknown. In the present research, genistein-resistant cells were induced. Gain- and loss-of-function studies of DEPTOR and ELK1 were performed in cells, and the cells were exposed to a condition with or without ELM to investigate the functions of ELK1, DEPTOR, and EKM in the sensitivity of PC cells to genistein.

## Materials and Methods

### Bioinformatics Analyses

A GSE97766 dataset comprising data of genistein-resistant and parental PC cells was downloaded from the Gene Expression Omnibus (GEO) database. The data were adjusted and analyzed using the Limma R Package. Differentially expressed genes between drug-resistant and parental PC cells were identified using ∣Fold Change∣ ≥ 2 and *p*- value < 0.05 as the thresholds. A gene set variant analysis (GSVA) was conducted based on the differentially expressed genes to identify the aberrantly activated signaling pathways. The GSVA was performed with the 50 gene sets from the h.all.v7.0.symbols.gmt of the Molecular Signatures Database (MSigDB) as the background. The activity of the signaling pathways in each sample was scored. Potential target genes of genistein were predicted on the Swiss Target Prediction system.^1^ The transcription factors binding to the promoter of DEPTOR were predicted on JASPAR.^2^

### Cell Culture

Human PC cell lines Panc-1 and PaCa were procured from Shanghai Institute of Biochemistry and Cell Biology, Chinese Academy of Sciences (Shanghai, China). Cells were cultured in Dulbecco’s Modified Eagle Medium containing 10% fetal bovine serum and 1% penicillin/streptomycin (Thermo Fisher Scientific, Inc., Waltham, MA, United States) at 37°C in humidified air enriched with 5% CO_2_. Exponentially growing cells were collected for the subsequent experiments.

### Cell Transfection

Overexpression vectors (oe) or short hairpin (sh) RNAs targeting DEPTOR and ELK1 were procured from GenePharma Co., Ltd (Shanghai, China). The recombinant vector and shRNA were used as negative control. All vectors and shRNAs were transfected into well growing parental or drug-resistant Panc-1 and PaCa cells using Lipofectamine 3000 Reagents (Invitrogen; Thermo Fisher Scientific, Carlsbad, CA, United States) according to the kit’s instructions.

### Construction of Genistein-Resistant Cell Lines

All cells were maintained in a 37°C incubator containing humidified air enriched with 5% CO_2_. Panc-1 and PaCa were continuously exposed to genistein (Sigma-Aldrich, St. Louis, MO, United States) to construct genistein-resistant cells. In brief, cells were exposed to different doses of genistein. Initially, the cells were exposed to 5 μM genistein for 48 h, once a week. When cells entered an exponential growth phase, the concentration of genistein was gradually increased till 100 μM. After 6 months of genistein treatment, the drug-resistant cells were sub-cultured and treated with 20 μM genistein every month to maintain the resistance. In the subsequent experiments, the genistein-resistant Panc-1 and PaCa cells were placed in a genistein-free condition for 14 days before use.

### CellTiter-Glo^®^ Luminescent Cell Viability Assay

Cells were cultured in 96-well microtiter plates at 5 × 10^3^ cells/well for the measurement of half maximal inhibitory concentration (IC50). After 48 h, the Panc-1 and PaCa cells were exposed to different doses of genistein (5, 10, 20, 40, 60, and 100 μM) for another 24 h. Then, 10 μl CTG solution was added into each well (Promega Corp., Madison, WI, United States) for 2 h of incubation in the dark. Then, the optical density (OD) of each well at 450 nm was examined by a microplate reader, and the genistein-induced reduction rate in cell viability was determined.

### Caspase-3 Activity Assay

Activity of caspase-3/7 in cells was detected utilizing a Caspase-Glo^®^ 3 Assay Kit (Promega) according to the kit’s protocols.

### Detection of the Lactate Dehydrogenase Release in Cells

Parental or drug-resistant cells were cultured in 96-well plates at 5 × 10^3^ cells/well for 48 h. Then, the cells were centrifuged at 350 *g* at 4°C for 5 min with the supernatant discarded. Thereafter, each well was added with 150 μl lactate dehydrogenase (LDH) release reagent (Cat. No. C0016; Beyotime Biotechnology, Shanghai, China) for 1 h of incubation at 37°C. The OD value at 490 nm was examined by the microplate reader to evaluate the level of LDH release in cells.

### Flow Cytometry

Annexin V and DNA staining was used to measure the apoptosis rate of cells during the process of genistein treatment (20 μM for continuous 48 h). The Annexin V was stained by fluorescein isothiocyanate, while the nucleic acid was stained by propidium iodide. The fluorescence was examined by a LSRFortessa flow cytometer (BD Biosciences, San Jose, CA, United States) and analyzed using the FlowJo software (Tree Star, Ashland, OR, United States).

### Colony Formation Assay

Cells were cultured in a condition with or without ELM treatment for 48 h and exposed to 20 μM genistein. After genistein treatment, the cells were immediately seeded in fresh medium at 200 cells per dish (60 mm) for 15 days. After that, the cells were fixed in formaldehyde and labeled with 0.1% crystal violet. The number of cell colonies (over 50 cells) was counted under a stereomicroscope (Olympus Optical, Tokyo, Japan).

### Immunofluorescence Staining

Once reaching a 60% cell confluence, the cells were fixed with 4% paraformaldehyde and in cold 100% methanol. Then, the cells were incubated with the mouse monoclonal antibody anti-mTOR (phospho S2448; 1:1,000, ab109268; Abcam Inc., Cambridge, MA, United States) at room temperature, and then with rabbit monoclonal Alexa Fluor 488 (1:750; Santa Cruz Biotechnology, Inc., Santa Cruz, CA, United States) for 60 min. The nuclei were stained with 4 ′, 6-diamidino-2-phenylindole.

### Reverse Transcription Quantitative Polymerase Chain Reaction

Total RNA from the transfected cells was extracted using the TRIzol reagent^®^ (Ambion, Thermo Fisher Scientific). The extracted mRNA was quantified using a ND-2000 spectrophotometer (Thermo Fisher Scientific). Then, 1 μg of RNA sample was reversely transcribed to cDNA utilizing a ReverTra Ace qPCR RT Kit (Toyobo Life Science, Osaka, Japan). Next, real-time qPCR was conducted using a SYBR^®^ RT-PCR kit on the an ABI7500 fast real-time PCR system (Applied Biosystems; Thermo Fisher Scientific). The primers used were acquired from Thermo Fisher Scientific. Gene expression was quantified using the 2^–ΔΔ*Ct*^ method with GAPDH serving as the internal loading control.

### Western Blot Analysis

Cells were lysed in RIPA buffer containing fresh protease inhibitor (Beyotime). The protein concentration was detected using a bicinchoninic acid kit (Beyotime). Next, an equal amount of protein sample was run on 10% SDS-PAGE and transferred to PVDF membranes (Thermo Fisher Scientific). After being blocked by 5% non-fat milk for 1 h, the membranes were cultured with the antibodies against DEPTOR (1:1,000, ab171944, Abcam), ELK1 (1:1,000, ab32042, Abcam), anti-mTOR (phospho S2448; 1:1,000, ab109268, Abcam) and GAPDH (1:5,000, #5174; CST, Beverly, MA, United States) at 4°C overnight. Then, the membranes were further co-cultured with HRP-labeled goat anti-rabbit (1:5,000, ab205718, Abcam) at 37°C for 1 h. The blots were visualized utilizing an enhanced chemiluminescence kit (WP20005, Invitrogen). Relative protein expression was assessed by Image-Pro Plus 6.0.

### Xenograft Tumors in Nude Mice

The animal study was performed based on a previous report (Ma et al., 2019). NOD/SCID nude mice (4 weeks old, 20 ± 2 g) were acquired from the SLAC Laboratory Animal Co., Ltd. (Shanghai, China). To explore the function of genistein on the growth of xenograft tumors *in vivo*, a 100 μl suspension of parental and drug-resistant cells (1 × 10^6^) with stable transfection was implanted into mice through subcutaneous injection (*n* = 5 in each group). From the 12th day after cell implantation, the mice were intraperitoneally injected with genistein once every 3 days for a total of three times (3 mg/kg each time). ELM was administrated in to mice at a 3-day interval as well through intragastric administration, while the control mice were given an equal volume of normal saline. Then, the volume (V) of tumors was determined routinely as follows: *V* = 0.5 × L × W^2^, where “L” indicates the length while “W” indicates the width of tumor. On the 35th day after cell implantation, the mice were euthanized by 150 mg/kg of pentobarbital sodium (intraperitoneal injection). Then, the tumors were taken out and weighed, and the tumor tissues were fixed and embedded in paraffin for the following histological staining. All animal studies were ratified by the Ethical Committee on Animal Research of Zhongshan Hospital. Great attempts were made to reduce the pain of animals. During the animal procedures, if the animals showed conditions such as a significant decline in body weight (>10%), piloerection, catalepsy, dyspnea, sialorrhea, tremor, spasm, or falling a dead sleep were, an appropriate amount of anesthetic or analgesic was given, such as 0.02 mg/kg of atropine or 30 mg/kg of ketamine. If the decline in body weight exceeded by over 25% or the spasm lasted for over 10 min, or the mice slept for over 1 h, the animals were euthanized using 150 mg/kg of pentobarbital sodium. After 30 min, the animal death was confirmed by the loss of neural reflex and link reflex, unresponsiveness to stimuli, and the loss of heartbeat rather than simple cardiac arrest.

### Terminal Deoxynucleotidyl Transferase-Mediated dUTP Nick End Labeling Assay

The tissues were cut into sections, dewaxed, and rehydrated, and terminal deoxynucleotidyl transferase dUTP nick end labeling (TUNEL) assay was performed to examine the cell apoptosis rate in the tissues. The TUNEL assay was conducted as previously reported (Kyrylkova et al., 2012).

### Immunohistochemical Staining

Immunohistochemical (IHC) staining was carried out according to a previous study (Ma et al., 2019). In brief, the paraffin-embedded sections (4 μm) were dewaxed and rehydrated. After antigen retrieval in citrate buffer, the sections were blocked in 100% normal goat serum and then incubated with anti-Ki67 (ab92742, 1:500, Abcam) at 4°C overnight, and then with HRP-conjugated goat anti-rabbit (1:1, Cat No. KIT-5905; Maixin Biotechnology, Fujian, China) at 37°C for 2 h. After that, the sections were counter-stained with hematoxylin and observed under the microscope.

### Luciferase Assay

The wild-type (WT) sequence containing the putative binding site with the DEPTOR promoter was obtained and amplified from the human genome DNA using PCR. The sequence was inserted into the downstream of the pGL3-promoter luciferase vector to construct recombinant pGL3-enhancer luciferase reporter vector. Then, the recombinant vector was co-transfected with oe-ELK3 into 293T cells using the Lipofectamine TM 2000 kit (Life Technologies, Carlsbad, CA, United States). After 24 h, the luciferase activity in cells was evaluated using a dual-luciferase-reporter-gene detection system (Promega).

### Chromatin Immunoprecipitation-qPCR

A chromatin Immunoprecipitation (ChIP) assay kit (Cat No. #53008; Active Motif, Carlsbad, CA, United States) was used according to the kit’s instructions. The cells were cross-linked in 1% formaldehyde at 20°C for 10 min and neutralized by glycine for 5 min. Next, the cells were washed in PBS and resuspended in SDS, followed by ultrasonic treatment. After further centrifugation, the supernatant was collected and diluted in immunoprecipitation dilution buffer. Anti-ELK1 (CST) and the control anti-IgG (Cat No. #2729S, CST) was applied for immunoprecipitation reaction. Then, the precipitates were incubated with cytarabine for 1 h. Next, the precipitates were washed and de-crosslinked, and the DNA was purified for qPCR.

### Statistical Analysis

Graphpad prism software (version 8; GraphPad Software, San Diego, CA, United States) was applied for data analysis. Differences between two groups were compared using the Student’s *t-* test, while those among multiple groups were analyzed by one-way or two-way analysis of variance (ANOVA) followed by Tukey’s multiple comparison. Data were collected from three independent experiments and presented as the mean ± standard deviation (SD). *p* < 0.05 represents significant difference.

## Results

### DEPTOR Expression Is Significantly Decreased in the Genistein-Resistant Cells

**FIGURE 1:**
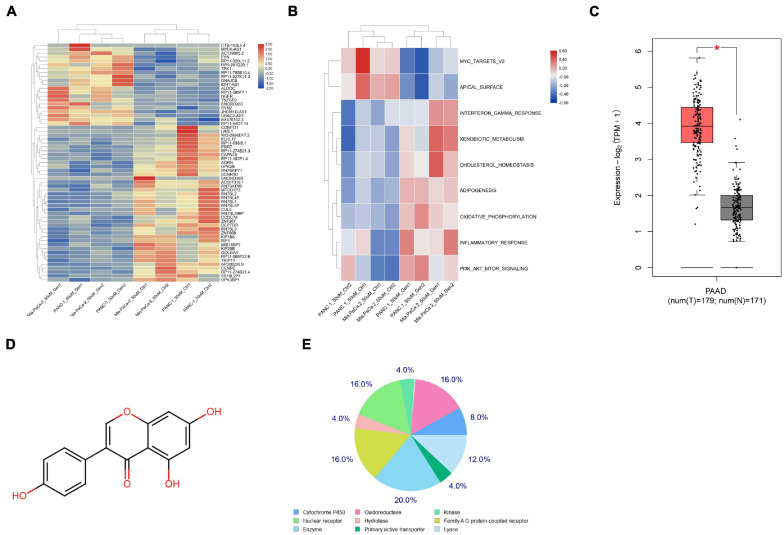
DEPTOR expression is notably decreased in the genistein-resistant cells. **(A)** A heatmap for differentially expressed mRNAs between drug-resistant and drug-sensitive cells according to a GEO GSE97766 dataset. **(B)** Aberrantly activated signaling pathway according to the differentially expressed mRNAs analyzed through GSVA. **(C)** DEPTOR expression predicted on the TCGA-GTEx Database; **(D)** the chemical structure of genistein. **(E)** Candidate target genes of genistein predicted using the Swiss Target Prediction system. Data were collected from three independent experiments and presented as mean ± SD. *p < 0.05.

To explore the molecules responsible for genistein-resistance in PC cells, we first obtained a GEO GSE97766 dataset comprising data from genistein-resistant and parental PC cells. The Limma R Package analyzed that DEPTOR was significantly downregulated in the drug-resistant cells (Figure 1A). Next, a GSVA was further performed based on the identified genes, and the activity of the PI3K/AKT-mTOR signaling pathway was found to be increased significantly (Figure 1B). According to the data in The Cancer Genome Atlas (TCGA)-Genotype-Tissue Expression (GTEx) Database, DEPTOR was predicted to be highly expressed in tumor samples (Figure 1C). Next, we explored the candidate targeting genes of genistein using the Swiss Target Prediction system. It was predicted that genistein can directly suppress EGFR (Figures 1D,E; Supplementary Table 1). However, EGFR has been reported to be negatively correlated with DEPTOR expression and activate the mTOR signaling pathway (Zhou et al., 2016). We therefore speculated that the EGFR was no longer suppressed by genistein in genistein-resistant cells, which blocks DEPTOR expression, therefore activating the PI3K/AKT/mTOR axis and increasing the drug resistance of PC cells.

### Upregulation of DEPTOR Promotes Sensitivity of PC Cells to Genistein

**FIGURE 2:**
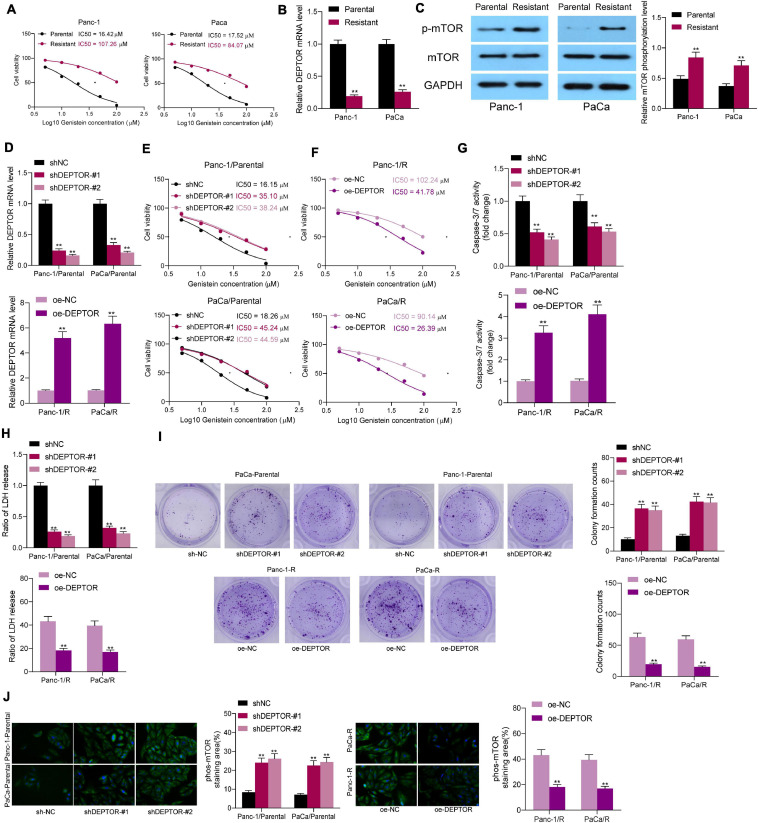
Upregulation of DEPTOR promotes sensitivity of PC cells to genistein. **(A)** IC50 of genistein in parental and drug-resistant Panc-1 and PaCa cells determined using a CTG kit. **(B)** mRNA expression of DEPTOR in cells evaluated by reverse transcription quantitative polymerase chain reaction (RT-qPCR). **(C)** Phosphorylation of mTOR in Panc-1 and PaCa cells detected by Western blot assay. **(D)** DEPTOR expression in parental and genistein-resistant Panc-1 and PaCa cells after DEPTOR interventions examined by RT-qPCR. **(E,F)** IC50 of genistein in parental and genistein-resistant cells determined using a CTG kit. **(G)** Activity of caspase-3/7 in cells after 20 μM genistein treatment determined using a caspase-3 kit. **(H)** Lactate dehydrogenase (LDH) release in cells after 20 μM genistein treatment determined using an LDH release kit. **(I)** Viability of cells after 20 μM genistein treatment evaluated using the colony formation assay. **(J)** mTOR phosphorylation in cells evaluated by Western blot analysis. Data were collected from three independent experiments and presented as mean ± SD. **p < 0.01.

To validate this, the parental Panc-1 and PaCa cells were treated with an ascending doses of genistein to induce drug-resistant cells. After 6 months, the IC50 of genistein in cells was examined using a CTG kit. It was found that the genistein-resistant cells were successfully established since the drug resistance of cells was significantly increased following serial genistein treatment (Figure 2A). Next, the mRNA expression of DEPTOR was obviously decreased in the genistein-resistant cell lines (Figure 2B), and the mTOR activity, correspondingly, was increased (Figure 2C).

To validate the role of DEPTOR in the sensitivity of PC cells to genistein, downregulation of DEPTOR was introduced in parental cells through administration of sh-DEPTOR, and upregulation of DEPTOR was induced in genistein-resistant cell lines through administration of oe-DEPTOR. The transfection efficacy was validated by reverse transcription quantitative polymerase chain reaction (RT-qPCR) (Figure 2D). Thereafter, the IC50 of genistein in cells was examined. Downregulation of DEPTOR in parental Panc-1 and PaCa cells decreased the sensitivity of cells to genistein (Figure 2E), whereas upregulation of DEPTOR in genistein-resistant Panc-1 and PaCa cells reduced the IC50 of genistein (Figure 2F). The IC50 of genistein in parental PC cells was approximately 20 μM. Therefore, cells were treated with 20 μM genistein in subsequent experiments to avoid a too drastic cytotoxicity on parental cells. After that, the activity of caspase-3/7 in cells was measured. It was found that the caspase-3/7 activity in parental cells was significantly decreased upon DEPTOR knockdown, while overexpression of DEPTOR increased the caspase-3/7 activity in the genistein-resistant cells (Figure 2G). In addition, the cytotoxicity of genistein in cells was further evaluated using an LDH kit. Likewise, the LDH release in parental cells was decreased after DEPTOR silencing, but it was increased in genistein-resistant cells upon DEPTOR upregulation (Figure 2H). Moreover, the viability of cells after genistein treatment was detected using a colony formation assay. It was found that the number of cell colonies formed by parental cells was increased after DEPTOR inhibition. Again, the number of colonies formed by genistein-resistant cells was decreased after DEPTOR overexpression (Figure 2I). The phosphorylation of mTOR in cells was further detected by immunofluorescence staining. As expected, DEPTOR negatively regulated the mTOR phosphorylation in cells according to the Western blot assays (Figure 2J).

### Administration of ELM Increases Sensitivity of PC Cells to Genistein

**FIGURE 3:**
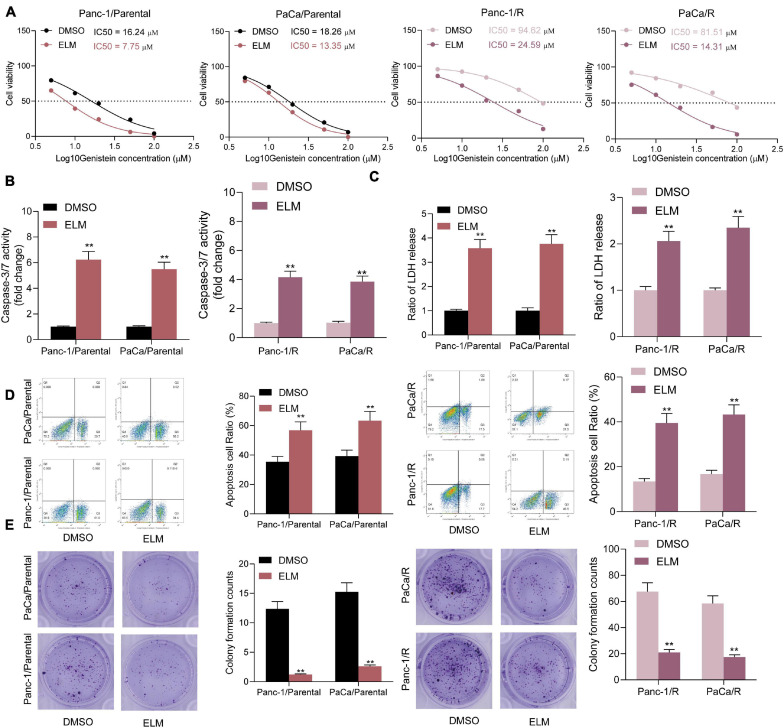
Administration of ELM increases sensitivity of PC cells to genistein. **(A)** IC50 of genistein in parental and genistein-resistant cells determined by a CTG kit. **(B)** Activity of Caspase-3/7 in cells examined by a caspase-3 kit. **(C)** Lactate dehydrogenase (LDH) release in cells detected using an LDH release kit. **(D)** Apoptosis rate in PC cells detected using flow cytometry. **(E)** Viability of cells evaluated using the colony formation assay. Data were collected from three independent experiments and presented as mean ± SD. **p < 0.01.

Following the findings above that mTOR activation was closely correlated with the genistein resistance in PC cells, a commercial mTOR-specific inhibitor ELM was used to treat the cells. After ELM administration, the IC50 of genistein in both parental and drug-resistant cells was significantly decreased (Figure 3A). Next, ELM was co-administrated with genistein in parental and genistein-resistant cells, after which the caspase-3 activity (Figure 3B), the LDH release (Figure 3C) and the apoptosis rate (Figure 3D) in cells were significantly increased. Moreover, the colony formation assay indicated that administration of ELM strengthened the suppressive effect of genistein on the colony formation ability of PC cells (Figure 3E).

### Administration of ELM Increases Sensitivity of PC Cells to Genistein *in vivo*

**FIGURE 4:**
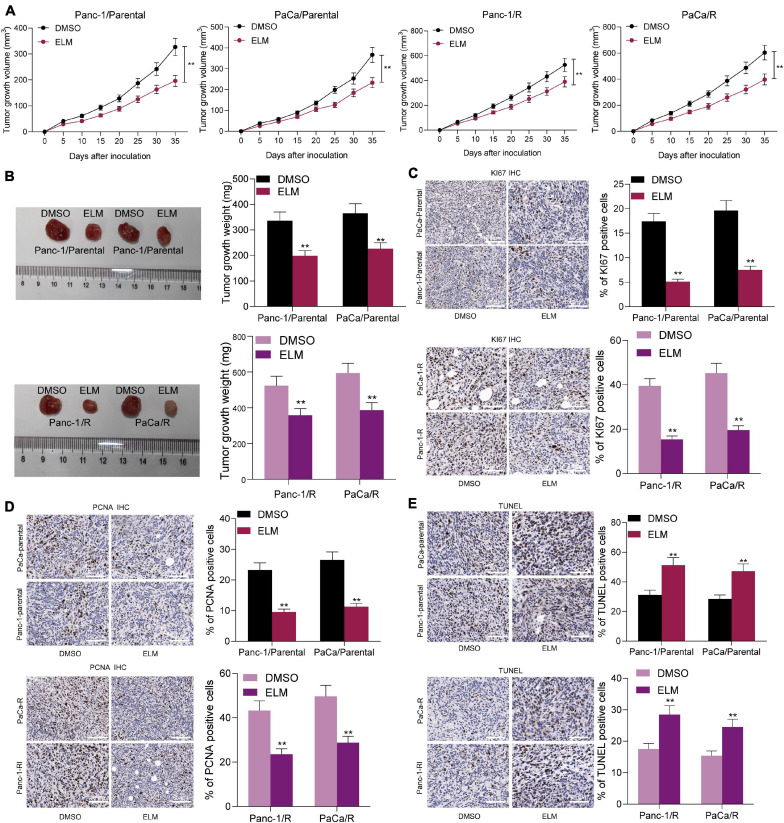
Administration of ELM increases sensitivity of PC cells to genistein. **(A)** Volume change of the xenograft tumors. **(B)** Weight of xenograft tumors on the 35th day. **(C,D)**, Staining intensity of KI67 **(C)** and PCNA **(D)** in tumor tissues determined by IHC staining. **(E)** Cell apoptosis in the tumor tissues measured by TUNEL assay. Data were collected from three independent experiments and presented as mean ± SD. **p < 0.01.

To further evaluate the relevance of ELM to the sensitivity of genistein on the growth of PC cells, the parental and genistein-resistant PaCa and Panc-1 cells were implanted into NOD/SCID mice for *in vivo* experiments. The mice were given genistein through intraperitoneal injection and given ELM through intragastric administration. The volume of xenograft tumor in mice was detected. The growth rate of xenograft tumor in mice was decreased after ELM administration (Figures 4A,B). In addition, the tumor tissues were collected for histological staining. The IHC staining results showed that the staining intensity of KI67 and PCNA in the tumor tissues was significantly decreased after ELM treatment (Figures 4C,D). In addition, the TUNEL assay suggested that the cytotoxicity of genistein to PC cells was strengthened by ELM (Figure 4E).

### ELK1 Is Highly Expressed in Genistein-Resistant Cells and Suppresses DEPTOR Transcription

**FIGURE 5:**
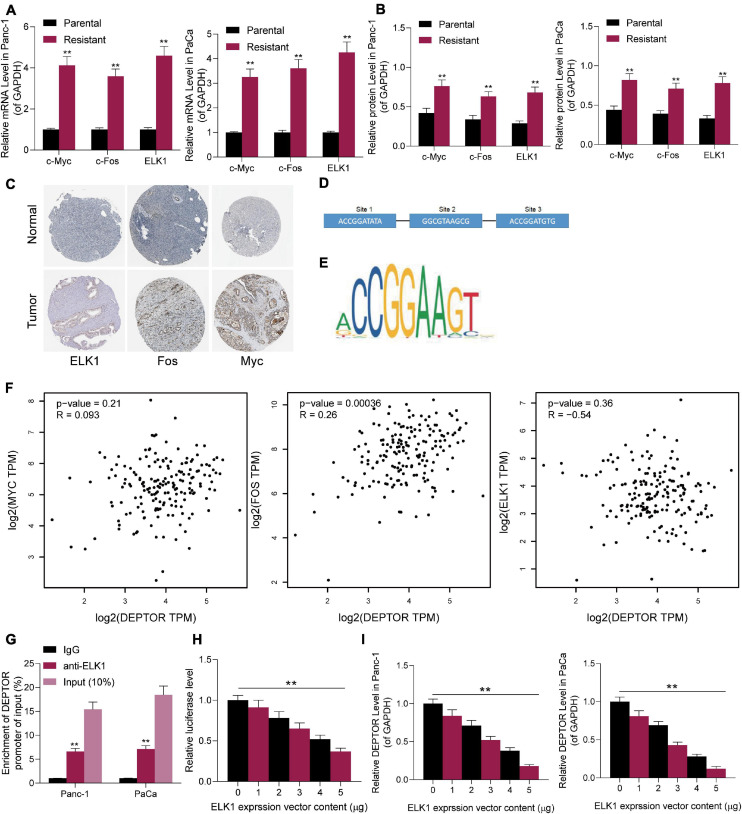
ELK1 is highly expressed in genistein-resistant cells and suppresses DEPTOR transcription. **(A,B)** mRNA **(A)** and protein **(B)** expression of c-Fos, c-Myc, and ELK1 in parental and genistein-resistant Panc-1 and PaCa cells examined by reverse transcription quantitative polymerase chain reaction (RT-qPCR) and Western blot assays, respectively; **(C)** Staining intensity of c-Fos, c-Myc, and ELK1 in PC tissues predicted on the Human Protein Atlas website. **(D)** Binding sites between c-Fos/c-Myc/ELK1 and DEPTOR in cells predicted on the JASPAR system. **(E)** The conservative binding sequence of ELK1. **(F)** Correlation between the expression of c-Fos/c-Myc/ELK1 and the expression DEPTOR in the TCGA-PAAD database using the GEPIA system. **(G,H)** Binding relationship between ELK1 and the promoter sequence of DEPTOR validated through ChIP-qPCR **(G)** and luciferase **(H)** assays. **(I)** mRNA expression of DEPTOR in Panc-1 and PaCa cells examined by RT-qPCR. Data were collected from three independent experiments and presented as mean ± SD. **p < 0.01.

The findings of bioinformatics analyses above suggested that genistein might target EGFR (Figures 1D,E; Supplementary Table 1). As mentioned above, we speculated that the inhibitory effect of genistein on EGFR might be blocked in drug-resistant cells. To validate this, we determined the expression of downstream transcription factors of this pathway in the parental and drug-resistant cells. The expression of c-Fos, c-Myc, and ELK1 in genistein-resistant cells was significantly elevated (Figures 5A,B). Subsequently, the data on the Human Protein Atlas website^3^ suggested that the staining intensity of c-Fos, c-Myc, and ELK1 in PC tissues was higher than that in normal pancreatic tissues (Figure 5C). To determine possible regulators of DEPTOR, we searched the JASPAR system to explore if there are binding relationships between these candidate transcription factors and DEPTOR. Consequently, ELK1 was predicted as the only one that owns putative binding sites with the DEPTOR promoter region (Figures 5D,E). In addition, we determined the correlation between c-Fos, c-Myc and ELK1, and DEPTOR in The Cancer Genome Atlas-Pancreatic adenocarcinoma (TCGA-PAAD) database using the GEPIA website.^4^ Also, ELK1 showed a high and negative correlation with DEPTOR expression (Figure 5F). To further validate the binding relationship between ELK1 and DEPTOR, a ChIP-qPCR assay was performed. An abundance of DEPTOR fragments was found in the precipitates pulled down by anti-ELK1 compared to anti-IgG (Figure 5G). Then, a luciferase assay was conducted where pGL3-enhancer luciferase reporter vectors containing the promoter sequence of DEPTOR were co-transfected with oe-ELK1 into 293T cells. The luciferase activity in cells was decreased with the increase of ELK1 concentration (Figure 5H). Further, Panc-1 and PaCa cells were transfected with oe-ELK1 as well, after which the expression of DEPTOR was decreased accordingly (Figure 5I). These findings suggested that ELK1 suppresses DEPTOR transcription. ELK1 is downstream the MAPK kinase pathway. Therefore, to examine whether the MAPK pathway is involved in the genistein resistance in PC cells, a MEK inhibitor trametinib (TMT) was administrated into the PC cells. After MEK inhibition, it was found that the sensitivity of both parental or drug-resistant cells to genistein was enhanced (Supplementary Figure 1).

### Overexpression of ELK1 Reduces Sensitivity of PC Cells to Genistein

**FIGURE 6:**
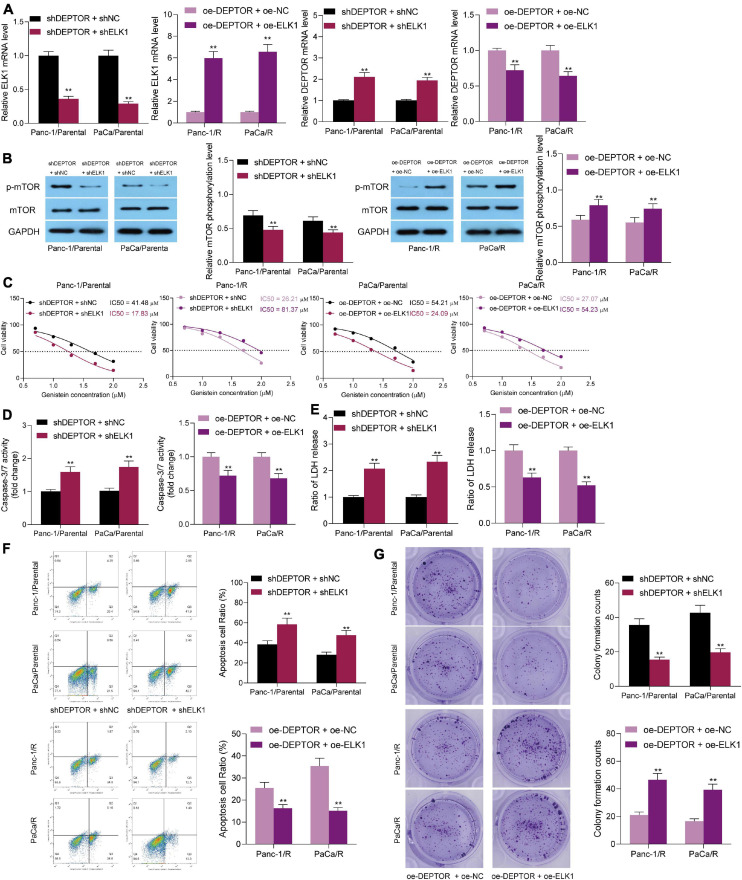
Overexpression of ELK1 reduces sensitivity of PC cells to genistein. **(A,B)** Protein level of ELK1 and phosphorylation of mTOR in genistein-resistant and parental cells examined by Western blot analysis. **(C)** IC50 of genistein in PC cells evaluated by a CTG kit. **(D)** Activity of caspase-3/7 in cells examined by a caspase-3 kit. **(E)** Lactate dehydrogenase (LDH) release in cells measured using an LDH release kit; **(F)** Apoptosis rate in PC cells assessed using flow cytometry. **(G)** Viability of cells evaluated using the colony formation assay. Data were collected from three independent experiments and presented as mean ± SD. **p < 0.01.

Next, we further verified the relevance of ELK1 expression to the genistein resistance in cells. Knockdown of ELK1 was introduced in parental PC cells pre-transfected with sh-DEPTOR, and upregulation of ELK1 was administrated in drug-resistant cells pre-transfected with oe-DEPTOR. Then, it was observed that the abundance of DEPTOR in parental cells was increased and the mTOR phosphorylation was decreased after ELK1 knockdown. By contrast, further overexpression of ELK1 reduced DEPTOR expression and enhanced phosphorylation of mTOR in drug-resistant cells (Figures 6A,B). Accordingly, the IC50 of genistein in parental cell lines was enhanced after ELK1 downregulation, whereas that in genistein-resistant cells was significantly enhanced upon ELK1 overexpression (Figure 6C). The caspase-3 activity and LDH release in parental cells were increased after ELK1 overexpression, but inverse trends were observed in the drug-resistant cells overexpressing ELK1 (Figures 6D,E). Likewise, the flow cytometry results suggested that the number of apoptotic cells induced by genistein was increased by sh-ELK1 in parental cells but reduced by oe-ELK1 in drug-resistant cells (Figure 6F). The number of cell colonies, according to the colony formation assay, was reduced after ELK1 downregulation but increased after ELK1 overexpression (Figure 6G).

### Overexpression of ELK1 Blocks the Promoting Role of ELM in the Genistein-Sensitivity in PC Cells

**FIGURE 7:**
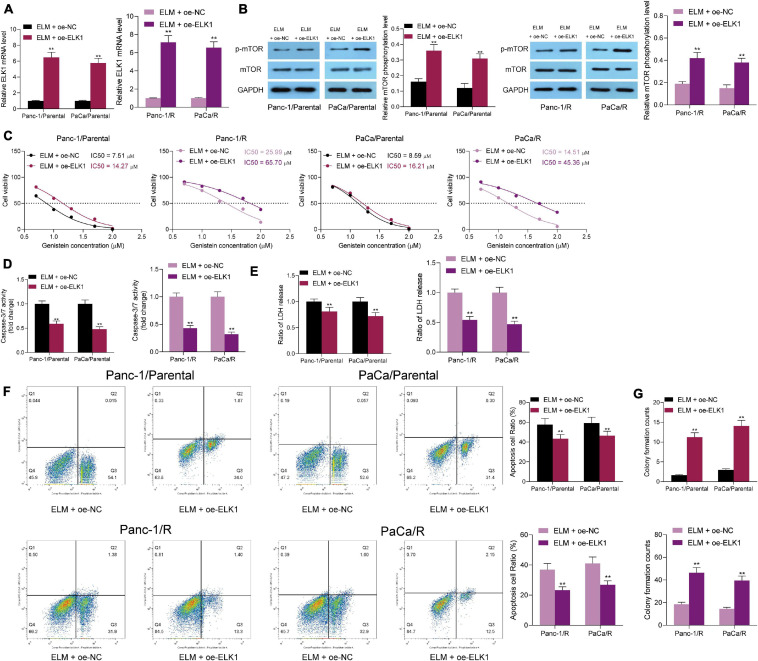
Downregulation of ELK1 blocks the promoting role of ELM in the genistein-sensitivity in PC cells. **(A)** Expression of ELK1 in PC cells after ELK1 interventions detected by RT-qPCR. **(B)** mTOR phosphorylation in cells determined by Western blot analysis. **(C)** IC50 of genistein in cells evaluated by a CTG kit. **(D)** Activity of caspase-3/7 in cells examined by a caspase-3 kit. **(E)** Lactate dehydrogenase (LDH) release in cells measured using an LDH release kit. **(F)** Apoptosis rate in PC cells assessed using flow cytometry. **(G)** Viability of cells evaluated using the colony formation assay. Data were collected from three independent experiments and presented as mean ± SD. **p < 0.01.

Since ELM was found to suppress the mTOR activity in cells, ELK1 was found to trigger mTOR phosphorylation through transcriptionally suppressing DEPTOR. To validate the molecular mechanism, the parental and drug-resistant Panc-1 and PaCa cells co-treated with ELM and genistein were further administrated with ELK1 overexpression vector. The transfection efficacy was validated by RT-qPCR again (Figure 7A), and the mTOR phosphorylation in cells was increased (Figure 7B). After ELK1 overexpression, the sensitivity of cells to genistein increased by ELM was reduced (Figure 7C). Under this condition, it was found that the cell apoptosis, caspase-3 activity, and LDH release in cells were reduced (Figures 7D–F). In addition, the colony formation ability suppressed by ELM was recovered upon ELK1 knockdown (Figure 7G). Collectively, these results indicated that downregulation of ELK1 blocks the promoting role of ELM in the genistein-sensitivity in PC cells.

## Discussion

Genomic changes are frequently involved in the onset and progression of cancers as well as the development of drug resistance to conventional chemotherapies. Comprehensive bioinformatic analyses are advanced and helpful tools to analyze gene expression and signaling pathways under different physiological and pathological conditions (Zhou et al., 2020). In the present work, we confirmed that downregulated DEPTOR induced by ELK1 is possibly involved in genistein resistance in PC cells. The integrated bioinformatic and experimental evidence suggested that concomitant administration of ELM or downregulation or ELK with genistein might strengthen the sensitivity of PC cells to genistein.

First, DEPTOR was predicted to be highly expressed in genistein-resistant cells using a GEO GSE97766 dataset, and the PI3K/Akt/mTOR signaling pathway was subsequently predicted to be activated. To validate this, genistein-resistant cells were induced, in which poor expression of DEPTOR and high phosphorylation of mTOR were observed. Poor expression of DEPTOR and activation of mTOR have been frequently found to be related to the drug resistance. For instance, DEPTOR was found to confer the sensitivity of the pituitary adenoma to dopamine agonist and block the proliferation of cells (Yao H. et al., 2019). Frequent activation of mTOR signaling in trastuzumab-resistant NCI N87 (T-R) HER2 + gastric cancer cells identified by a parallel quantitative proteomics profiling suggested the need of an mTOR antagonist for the T-R HER + gastric cancers (Liu et al., 2017). In line with this, activation of the Akt/mTOR signaling pathway has been reported to induce drug resistance and promote proliferation and migration of gastric cancer cells (Yao Y. et al., 2019). More relevantly, downregulation of the PI3K/Akt/mTOR pathway was documented to suppress cell death and chemosensitivity of PC cells (Lan et al., 2019). In our subsequent experiments, downregulation of DEPTOR was induced in parental PC cell lines while upregulation of DEPTOR was introduced in genistein-resistant cell lines. Consequently, DEPTOR overexpression largely enhanced the drug sensitivity of the resistant Panc-1 and PaCa cells and increased the cell apoptosis, while DEPTOR downregulation in parental cells led to inverse trends, indicating the correlation between DEPTOR inhibition and the genistein resistance in PC cells.

In a previous report by Lu et al. (2017) a gene set enrichment analysis identified the activation of mTOR signaling in oxaliplatin-treated early passaged cell lines and patient-derived xenografts of colon cancer, and co-treatment of oxaliplatin with an mTOR inhibitor demonstrated an additive effect in the *in vitro* and *in vivo* experiments. ELM is a well-established mTOR inhibitor that has been suggested as an ideal combination regimen with chemotherapy for the management of cancers (Li et al., 2016; Generali et al., 2017; Alves et al., 2019). A clinical trial study has also indicated that the combination of ELM and multi-agent chemotherapy was well-tolerated in patients with acute lymphoblastic leukemia (Place et al., 2018). Combination of ELM and gemcitabine has been found to overcome the resistance of PC cells to gemcitabine treatment through caspase-induced cell apoptosis and cell cycle arrest (Peng and Dou, 2017). In addition, oral ELM in combination with capecitabine treatment has shown moderate active efficacy against advanced PC (Kordes et al., 2015). Here, we validated that the administration of genistein strengthened the sensitivity of PC cells to genistein treatment, presenting as increased cell apoptosis, reduced cell proliferation, increased release of LDH, and caspase-3/activity in cells *in vivo*. Also, gavage administration of ELM reduced the volume and weight of tumors in animal experiments. These results validated that co-treatment of ELM with genistein is a promising treatment for the clinical management of PC.

Importantly, our bioinformatic analysis suggested that genistein could suppress EGFR. A similar trend has been witnessed in cholangiocarcinoma that genistein reduces the activation of AKT and EGFR according to a previous report by Tanjak et al. (2018). Synthetic genistein glycosides were also observed to suppress EGFR phosphorylation and enhance the efficacy of radiotherapy in colon cancer cells (Gruca et al., 2014). However, compared to the parental PC cells, increased expression of EGFR-related factors c-Fos, c-Myc, and ELK1 was observed in the genistein-resistant cells. We then supposed that the failure in EGFR suppression is accountable for the development of genistein resistance in PC cells since EGFR has been reported to lead to constitutive activation of its dependent signaling pathways, especially the PI3K/AKT/mTOR pathway (El Guerrab et al., 2020). Co-treatment of ELM has been reported to enhance the tumor-suppressing role of gefitinib, an EGFR antagonist, in EGFR WT lung cancer cells (Huang et al., 2013). Interestingly, inhibition of DEPTOR has been reported to be involved in EGFR-mediated mTOR signaling activation (Zhou et al., 2016). Subsequently, we validated a binding relationship between ELK1 and the promoter region of DEPTOR, and DEPTOR expression in PC cells was decreased as ELK1 abundance increased, indicating that the ELK1-mediated DEPTOR downregulation is potentially correlated with mTOR activation and the development of genistein resistance. Inactivation of ELK1 has been found to enhance the cytotoxic activity of a common chemo drug, cisplatin, to bladder cancer cells (Kawahara et al., 2015). Also, downregulation of ELK1 has been found to suppress the cancerogenesis of PC (Li et al., 2020). Here, we observed that the overexpression of ELK1 enhanced the resistance of PCa cells to genistein treatment. Also, upregulation of ELK1 blocked the sensitizing function of ELM in PC cells to genistein as well, indicating that downregulation of ELK1 is necessary for overcoming the genistein resistance in PC cells.

## Conclusion

**FIGURE 8:**
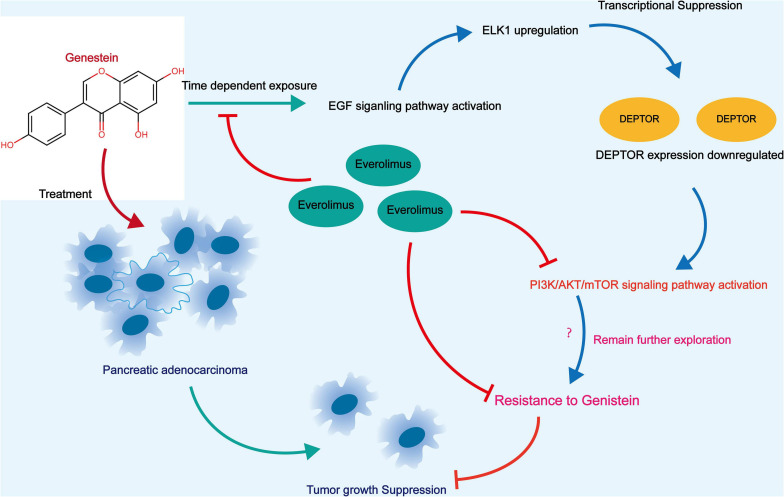
A graphical abstract for the molecular mechanism. In genistein-resistant PC cells, the suppressive effect of genistein on EGFR signaling was blocked. The downstream molecule ELK1 induces DEPTOR downregulation and enhances mTOR phosphorylation, which further leads to increased genistein resistance in cells. Treatment of ELM or artificial downregulation ELK1 may block the mTOR phosphorylation and enhance the genistein sensitivity in cells.

This study demonstrated that ELK1-induced DEPTOR downregulation may confer to genistein resistance to PC cells through activation of the mTOR signaling (Figure 8). The present study provided evidence that co-treatment with ELM, or downregulation of ELK1 can reduce mTOR phosphorylation and counteract the resistance of PC cells to genistein. We also hope more studies will be carried out to confirm our findings or to develop more thoughts in the management of this malignancy.

## Data Availability Statement

The original contributions presented in the study are included in the article/Supplementary Material, further inquiries can be directed to the corresponding author.

## Ethics Statement

The animal study was reviewed and approved by Ethical Committee on Animal Research of Zhongshan Hospital.

## Author Contributions

TL, TK, and ZY contributed to the original article and the experiment designing. TL, TK, ZY, QZ, WZ, and YF were devoted to the data and analysis. TL and YF wrote the manuscript including the design of figures. All authors approved the final manuscript.

## Conflict of Interest

The authors declare that the research was conducted in the absence of any commercial or financial relationships that could be construed as a potential conflict of interest.

## Publisher’s Note

All claims expressed in this article are solely those of the authors and do not necessarily represent those of their affiliated organizations, or those of the publisher, the editors and the reviewers. Any product that may be evaluated in this article, or claim that may be made by its manufacturer, is not guaranteed or endorsed by the publisher.

## Footnotes

1
http://www.swisstargetprediction.ch/

2
http://jaspar.genereg.net/

3
https://www.proteinatlas.org/

4
http://gepia.cancer-pku.cn/index.html
