# Supplementary material for: Co-treatment With Everolimus, an mTOR-Specific Antagonist, or Downregulation of ELK1 Enhances the Sensitivity of Pancreatic Cancer Cells to Genistein
Source: Front Cell Dev Biol. 2021 Sep 3;9:633035. doi: 10.3389/fcell.2021.633035 (PMC8448347; doi:10.3389/fcell.2021.633035)
Supplement: Supplementary file 1 [file Data_Sheet_1.docx]

**Supplementary Fig. S1**

**
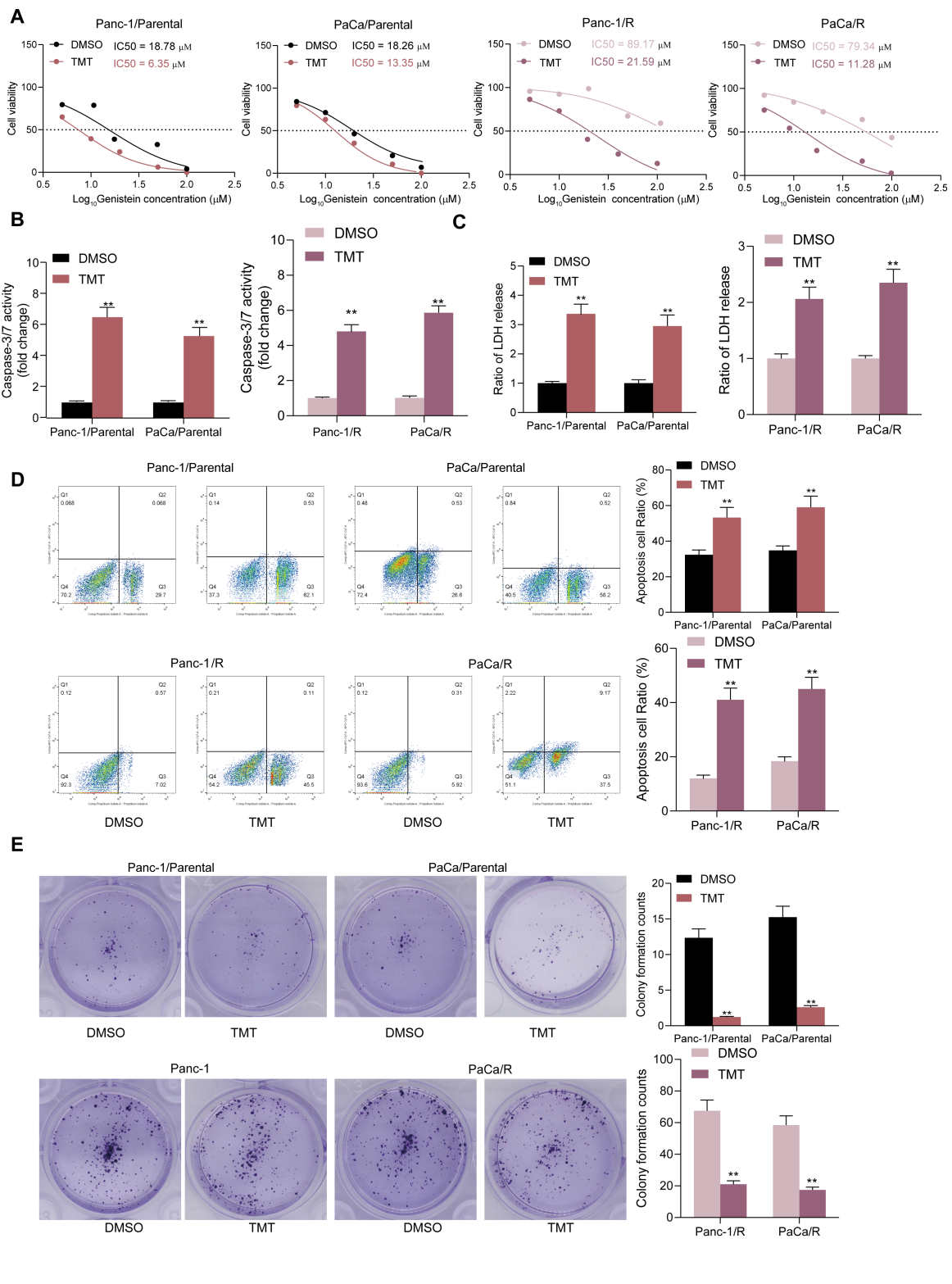
**

**Supplementary Fig. S1** TMT treatment enhances the sensitivity of both parental or drug-resistant cells to genistein. A, IC50 of genistein in PC cells evaluated by a CTG kit; B, activity of Caspase-3/7 in cells examined by a Caspase-3 kit; C, LDH release in cells measured using an LDH release kit; D, apoptosis rate in PC cells assessed using flow cytometry; E, viability of cells evaluated using the colony formation assay. Data were collected from three independent experiments and presented as mean ± SD. ***p* < 0.01.
