# Supplementary material for: Co-treatment With Everolimus, an mTOR-Specific Antagonist, or Downregulation of ELK1 Enhances the Sensitivity of Pancreatic Cancer Cells to Genistein
Source: Front Cell Dev Biol. 2021 Sep 3;9:633035. doi: 10.3389/fcell.2021.633035 (PMC8448347; doi:10.3389/fcell.2021.633035)
Supplement: Supplementary file 2 [file Data_Sheet_2.PDF]

SwissTargetPrediction

Target	Common name	Uniprot ID	ChEMBL ID	Target Class	Probability*	Known actives (3D/2D)
Thromboxane-A synthase	TBXAS1	P24557	CHEMBL1835	Cytochrome P450	1.0	1 / 2
Monoamine oxidase A	MAOA	P21397	CHEMBL1951	Oxidoreductase	1.0	6 / 34
Epidermal growth factor receptor erbB1	EGFR	P00533	CHEMBL203	Kinase	1.0	7 / 6
Estrogen receptor alpha	ESR1	P03372	CHEMBL206	Nuclear receptor	1.0	76 / 77
Maltase-glucoamylase	MGAM	O43451	CHEMBL2074	Hydrolase	1.0	1 / 1
Serotonin 2a (5-HT2a) receptor	HTR2A	P28223	CHEMBL224	Family A G protein-coupled receptor	1.0	1 / 2
Serotonin 2c (5-HT2c) receptor	HTR2C	P28335	CHEMBL225	Family A G protein-coupled receptor	1.0	1 / 3
Adenosine A1 receptor (by homology)	ADORA1	P30542	CHEMBL226	Family A G protein-coupled receptor	1.0	6 / 20
Estrogen receptor beta	ESR2	Q92731	CHEMBL242	Nuclear receptor	1.0	93 / 66
Adenosine A2a receptor	ADORA2A	P29274	CHEMBL251	Family A G protein-coupled receptor	1.0	5 / 15
Estradiol 17-beta-dehydrogenase 1	HSD17B1	P14061	CHEMBL3181	Enzyme	1.0	10 / 5
Estrogen-related receptor alpha	ESRRA	P11474	CHEMBL3429	Nuclear receptor	1.0	2 / 2
Estrogen-related receptor beta	ESRRB	O95718	CHEMBL3751	Nuclear receptor	1.0	1 / 1
ATP-binding cassette sub-family G member 2	ABCG2	Q9UNQ0	CHEMBL5393	Primary active transporter	1.0	6 / 27
Carbonic anhydrase VII	CA7	P43166	CHEMBL2326	Lyase	0.559044784358	8 / 12
Carbonic anhydrase XII	CA12	O43570	CHEMBL3242	Lyase	0.559044784358	10 / 18
Aldehyde dehydrogenase	ALDH2	P05091	CHEMBL1935	Oxidoreductase	0.54289985377	1 / 47
Carbonic anhydrase IV	CA4	P22748	CHEMBL3729	Lyase	0.54289985377	7 / 9
Cytochrome P450 19A1	CYP19A1	P11511	CHEMBL1978	Cytochrome P450	0.518401967066	6 / 27
Arachidonate 12-lipoxygenase	ALOX12	P18054	CHEMBL3687	Enzyme	0.444639417769	9 / 11
Tyrosinase (by homology)	TYR	P14679	CHEMBL1973	Oxidoreductase	0.420066875222	2 / 2
Macrophage migration inhibitory factor	MIF	P14174	CHEMBL2085	Enzyme	0.420066875222	1 / 8
Estradiol 17-beta-dehydrogenase 2	HSD17B2	P37059	CHEMBL2789	Enzyme	0.387283728398	10 / 4

Target	Common name	Uniprot ID	ChEMBL ID	Target Class	Probability*	Known actives (3D/2D)
Xanthine dehydrogenase	XDH	P47989	CHEMBL1929	Oxidoreductase	0.354464973753	12 / 20
6-phosphofructo-2-kinase/fructose-2,6-bisphosphatase 3	PFKFB3	Q16875	CHEMBL2331053	Enzyme	0.288928252457	2 / 2
Receptor-type tyrosine-protein phosphatase S	PTPRS	Q13332	CHEMBL2396508	Phosphatase	0.247942822879	6 / 7
P-glycoprotein 1	ABCB1	P08183	CHEMBL4302	Primary active transporter	0.231637184894	12 / 39
Arachidonate 15-lipoxygenase	ALOX15	P16050	CHEMBL2903	Enzyme	0.17427075329	6 / 8
NADPH oxidase 4	NOX4	Q9NPH5	CHEMBL1250375	Enzyme	0.157929217109	6 / 6
Carbonyl reductase [NADPH] 1	CBR1	P16152	CHEMBL5586	Enzyme	0.157929217109	2 / 2
Interleukin-2	IL2	P60568	CHEMBL5880	Secreted protein	0.149732593856	0 / 4
Cyclooxygenase-1	PTGS1	P23219	CHEMBL221	Oxidoreductase	0.108770969359	0 / 3
Norepinephrine transporter	SLC6A2	P23975	CHEMBL222	Electrochemical transporter	0.108770969359	0 / 1
Carbonic anhydrase II	CA2	P00918	CHEMBL205	Lyase	0.108770969359	8 / 22
Monoamine oxidase B	MAOB	P27338	CHEMBL2039	Oxidoreductase	0.108770969359	0 / 77
Acetylcholinesterase	ACHE	P22303	CHEMBL220	Hydrolase	0.100578902067	4 / 33
Arachidonate 5-lipoxygenase	ALOX5	P09917	CHEMBL215	Oxidoreductase	0.100578902067	4 / 19
Serum paraoxonase/ arylesterase 1	PON1	P27169	CHEMBL3167	Enzyme	0.100578902067	0 / 1
Carbonic anhydrase I	CA1	P00915	CHEMBL261	Lyase	0.100578902067	4 / 20
Cyclin-dependent kinase 5/CDK5 activator 1	CDK5R1 CDK5	Q15078 Q00535	CHEMBL1907600	Kinase	0.100578902067	6 / 6
Aldose reductase (by homology)	AKR1B1	P15121	CHEMBL1900	Enzyme	0.100578902067	16 / 44
Tankyrase-2	TNKS2	Q9H2K2	CHEMBL6154	Enzyme	0.100578902067	4 / 8
Tankyrase-1	TNKS	O95271	CHEMBL6164	Enzyme	0.100578902067	4 / 11
Tyrosine-protein kinase receptor FLT3	FLT3	P36888	CHEMBL1974	Kinase	0.100578902067	5 / 7
Cyclin-dependent kinase 1/cyclin B	CCNB3 CDK1 CCNB1 CCNB2	Q8WWL7 P06493 P14635 O95067	CHEMBL2094127	Other cytosolic protein	0.100578902067	4 / 4
Cyclooxygenase-2	PTGS2	P35354	CHEMBL230	Oxidoreductase	0.100578902067	1 / 8
Cyclin-dependent kinase 6	CDK6	Q00534	CHEMBL2508	Kinase	0.100578902067	3 / 3
Tyrosine-protein kinase SYK	SYK	P43405	CHEMBL2599	Kinase	0.100578902067	3 / 3
Glycogen synthase kinase-3 beta	GSK3B	P49841	CHEMBL262	Kinase	0.100578902067	3 / 7
Multidrug resistance-associated protein 1	ABCC1	P33527	CHEMBL3004	Primary active transporter	0.100578902067	7 / 10
Transthyretin	TTR	P02766	CHEMBL3194	Secreted protein	0.100578902067	2 / 2
Casein kinase II alpha	CSNK2A1	P68400	CHEMBL3629	Kinase	0.100578902067	3 / 3

Target	Common name	Uniprot ID	ChEMBL ID	Target Class	Probability*	Known actives (3D/2D)
Cystic fibrosis transmembrane conductance regulator	CFTR	P13569	CHEMBL4051	Other ion channel	0.100578902067	1 / 1
Cytochrome P450 1B1	CYP1B1	Q16678	CHEMBL4878	Cytochrome P450	0.100578902067	12 / 24
Aldo-keto reductase family 1 member B10	AKR1B10	O60218	CHEMBL5983	Enzyme	0.100578902067	2 / 3
Steryl-sulfatase	STS	P08842	CHEMBL3559	Enzyme	0.100578902067	0 / 6
Poly [ADP-ribose] polymerase-1	PARP1	P09874	CHEMBL3105	Enzyme	0.100578902067	3 / 6
Insulin-like growth factor binding protein 3	IGFBP3	P17936	CHEMBL3997	Secreted protein	0.100578902067	2 / 0
Alpha-synuclein	SNCA	P37840	CHEMBL6152	Unclassified protein	0.100578902067	2 / 0
Induced myeloid leukemia cell differentiation protein Mcl-1	MCL1	Q07820	CHEMBL4361	Other cytosolic protein	0.100578902067	3 / 4
Toll-like receptor (TLR7/TLR9)	TLR9	Q9NR96	CHEMBL5804	Toll-like and Il-1 receptors	0.100578902067	0 / 1
Corticotropin releasing factor receptor 1	CRHR1	P34998	CHEMBL1800	Family B G protein-coupled receptor	0.100578902067	1 / 0
Peroxisome proliferator-activated receptor alpha	PPARA	Q07869	CHEMBL239	Nuclear receptor	0.100578902067	1 / 1
Protein-tyrosine phosphatase 1B	PTPN1	P18031	CHEMBL335	Phosphatase	0.100578902067	5 / 21
Serine/threonine-protein kinase PIM1	PIM1	P11309	CHEMBL2147	Kinase	0.100578902067	8 / 6
Serine/threonine-protein kinase Aurora-B	AURKB	Q96GD4	CHEMBL2185	Kinase	0.100578902067	3 / 3
Tissue-type plasminogen activator	PLAT	P00750	CHEMBL1873	Protease	0.100578902067	8 / 0
Thrombin and coagulation factor X	F10	P00742	CHEMBL244	Protease	0.100578902067	10 / 0
Urokinase-type plasminogen activator	PLAU	P00749	CHEMBL3286	Protease	0.100578902067	13 / 0
Carbonic anhydrase III	CA3	P07451	CHEMBL2885	Lyase	0.100578902067	1 / 3
Carbonic anhydrase VI	CA6	P23280	CHEMBL3025	Lyase	0.100578902067	1 / 3
Carbonic anhydrase XIV	CA14	Q9ULX7	CHEMBL3510	Lyase	0.100578902067	1 / 3
Carbonic anhydrase IX	CA9	Q16790	CHEMBL3594	Lyase	0.100578902067	3 / 15
Carbonic anhydrase XIII	CA13	Q8N1Q1	CHEMBL3912	Lyase	0.100578902067	1 / 4
Carbonic anhydrase VA	CA5A	P35218	CHEMBL4789	Lyase	0.100578902067	1 / 3
Insulin-like growth factor binding protein 6	IGFBP6	P24592	CHEMBL2139	Secreted protein	0.100578902067	1 / 0

Target	Common name	Uniprot ID	ChEMBL ID	Target Class	Probability*	Known actives (3D/2D)
Insulin-like growth factor binding protein 4	IGFBP4	P22692	CHEMBL2310	Secreted protein	0.100578902067	1 / 0
Insulin-like growth factor binding protein 5	IGFBP5	P24593	CHEMBL2665	Secreted protein	0.100578902067	1 / 0
Insulin-like growth factor binding protein 2	IGFBP2	P18065	CHEMBL3088	Secreted protein	0.100578902067	1 / 0
Insulin-like growth factor binding protein 1	IGFBP1	P08833	CHEMBL4178	Secreted protein	0.100578902067	1 / 0
Arginase-1 (by homology)	ARG1	P05089	CHEMBL1075097	Enzyme	0.100578902067	2 / 2
Bcl2-antagonist of cell death (BAD)	BAD	Q92934	CHEMBL3817	Other cytosolic protein	0.100578902067	0 / 1
Glyoxalase I	GLO1	Q04760	CHEMBL2424	Enzyme	0.100578902067	3 / 3
Beta amyloid A4 protein	APP	P05067	CHEMBL2487	Membrane receptor	0.100578902067	2 / 3
Matrix metalloproteinase 9	MMP9	P14780	CHEMBL321	Protease	0.100578902067	2 / 2
Matrix metalloproteinase 2	MMP2	P08253	CHEMBL333	Protease	0.100578902067	2 / 2
Matrix metalloproteinase 12	MMP12	P39900	CHEMBL4393	Protease	0.100578902067	1 / 1
Lymphocyte differentiation antigen CD38	CD38	P28907	CHEMBL4660	Enzyme	0.100578902067	2 / 2
DNA topoisomerase I (by homology)	TOP1	P11387	CHEMBL1781	Isomerase	0.100578902067	1 / 1
Insulin-like growth factor I receptor	IGF1R	P08069	CHEMBL1957	Kinase	0.0	3 / 2
Vascular endothelial growth factor receptor 2	KDR	P35968	CHEMBL279	Kinase	0.0	2 / 2
Serine/threonine-protein kinase PLK1	PLK1	P53350	CHEMBL3024	Kinase	0.0	2 / 3
Hepatocyte growth factor receptor	MET	P08581	CHEMBL3717	Kinase	0.0	4 / 2
ALK tyrosine kinase receptor	ALK	Q9UM73	CHEMBL4247	Kinase	0.0	2 / 2
Tyrosine-protein kinase receptor UFO	AXL	P30530	CHEMBL4895	Kinase	0.0	2 / 2
Carbonic anhydrase VB	CA5B	Q9Y2D0	CHEMBL3969	Lyase	0.0	0 / 2
Protein-tyrosine phosphatase 4A3	PTP4A3	O75365	CHEMBL4162	Phosphatase	0.0	1 / 0
Peroxisome proliferator-activated receptor gamma	PPARG	P37231	CHEMBL235	Nuclear receptor	0.0	0 / 2
Protein farnesyltransferase	FNTA FNTB	P49354 P49356	CHEMBL2094108	Enzyme	0.0	1 / 0
Dopamine D3 receptor	DRD3	P35462	CHEMBL234	Family A G	0.0	1 / 1

Target	Common name	Uniprot ID	ChEMBL ID	Target Class	Probability*	Known actives (3D/2D)
				protein-coupled receptor		
